# *Bacillus* sp. SW14 isolated from arid mangroves sediments enhances tomato plant growth: insights from genome analysis and greenhouse evaluation

**DOI:** 10.3389/fpls.2025.1673790

**Published:** 2025-10-08

**Authors:** Balamurugan Sadaiappan, Qurban Ali, Mahideen Afridi, Munawwar Ali Khan, Sunil Mundra

**Affiliations:** ^1^ Khalifa Center for Genetic Engineering and Biotechnology (KCGEB), United Arab Emirates University, Al Ain, United Arab Emirates; ^2^ Department of Biology, College of Science, United Arab Emirates University, Al-Ain, Abu Dhabi, United Arab Emirates; ^3^ National Nanfan Research Institute, Chinese Academy of Agriculture Sciences, Sanya, China; ^4^ Department of Life and Environmental Sciences, College of Natural and Health Sciences, Zayed University, Dubai, United Arab Emirates; ^5^ National Water and Energy Center, United Arab Emirates University, Al Ain, United Arab Emirates

**Keywords:** mangrove, arid agricultural, *Solanum lycopersicum*, *Bacillus species*, plant growth-promoting bacteria, complete genome sequencing

## Abstract

Mangroves grow in arid regions with high temperatures, low precipitation, and evaporation. Despite low humidity and high salt concentration, these arid mangroves support diverse microorganisms, including salt-and temperature tolerant. The bacteria isolated from these arid mangroves have potential applications in arid agronomy, which alleviate plant stress and enhance plant growth. In this study, we characterize the genome of previously reported PGP and stress-tolerating bacteria, *Bacillus* sp. SW14, isolated from mangrove sediments of Umm Al Quwain, United Arab Emirates. Further, we investigated its effects on tomato seed germination and plant growth. Genome size of the strain SW14 is 4.25 MB, with a G+C content of 43.72%. Genome-based phylogenetic and ANI analyses confirmed strain SW14 as a novel *Bacillus* sp. The genome also consists of multiple PGP trait-related genes such as nitrogen fixation (*nifSUM*), ammonia production (*glnAB, ureABCDE*), phosphate (*pqq*, *PhoADEHPRU*, and *pstABCS*) and potassium solubilization (*kch/mthK, trkAGH* and *ktrAB*), Indole-3-acetic acid (IAA) (*trpABCDEFS*, *iaaT/yedL/ysnE* and *yhcx*) and siderophore (*dhbABCEF*) production. It also includes genes associated with salt and heat (*GrpE, dnaJ, K and groL, S*) stress tolerance and bactericidal and fungicidal genes. Greenhouse trial revealed that the inoculation of strain SW14 significantly improved tomato plant growth, increased leaf number (Control (C): 6.82 ± 3.21, Incubated (I): 20.51 ± 11.55), leaf fresh weight (C: 0.11 ± 0.044, I: 0.15 ± 0.0035), leaf surface area (C: 1.823 ± 0.144, I: 15.15 ± 4.13), shoot height (C: 4.56 ± 0.94, I: 10.54 ± 3.44), and average Soil-Plant Analysis Development (SPAD) values (C: 31.01 ± 1.46, I: 33.79 ± 1.68). Additionally, parameters such as chlorophyll a (C: 2.21 ± 0.84, I: 3.39 ± 0.51), total chlorophyll (a + b) (C: 3.33 ± 1.26, I: 4.91 ± 0.58), carotenoid (C: 0.81 ± 0.29, I: 4.70 ± 0.55) and total chlorophyll to carotenoid (C: 3.81 ± 1.38, I: 9.28 ± 1.28) in tomatoes inoculated with the strain SW14 were increased. In conclusion, *Bacillus* sp. SW14 is a promising candidate for supporting plant growth in arid agroecosystems since it possesses a variety of PGP genes and the ability to thrive in high temperatures and salinity conditions.

## Introduction

Mangroves aren’t limited to tropical and subtropical environments; they also grow in arid regions (latitude between 20 and 33 degrees north and south), such as the Middle East, the Gulf of California in Mexico, subtropical Africa, Western Australia, and Western South America. These arid mangroves adapt to high temperatures, withstand low precipitation and high evaporation caused by high solar radiation (Adame et al., 2021). Despite low humidity and high salt concentration, these arid mangroves support diverse microorganisms, including salt- and heat-tolerant strains. Studies have shown that bacteria isolated from the arid mangrove sediments and rhizosphere have multiple Plant Growth Promoting (PGP) traits (Afridi et al., 2024; Pallavi et al., 2023). These bacteria performing crucial functions like nitrogen fixation, phosphate (P) and potassium (K) solubilization and producing plant hormones like indole-3-acetic acid (IAA) (Widawati et al., 2022). For example, *Enterobacter cloacae* and *Kocuria rhizophila* isolated from *Avicennia marina* rhizosphere sediments can solubilize phosphate, produce IAA, and ammonia (Dey et al., 2024a). Similarly, *Rhodococcus* sp and *Arthrobacter* sp (Aruna et al., 2023) isolated from mangrove sediments, reported higher P solubilizing ability (Aruna et al., 2023; Afridi et al., 2024).

In addition to nutrient mobilization, PGPBs from mangrove environments can also resist and remove heavy metals (Dey et al., 2024b), which mitigate plant stress and improve plant growth, especially in arid environments, where drought is a significant factor affecting plant growth and productivity (Bodner et al., 2015). These adaptive traits of mangrove associated PGPBs are particularly important in the expansion of drylands under climate change. Due to climate change, over 50% of arable land may face challenges related to plant growth by 2050 (Mohanty et al., 2021). Approximately 40% of Earth’s surface already consist drylands-including arid, semi-arid, and hyper-arid lands (Feng and Fu, 2013; Middleton and Thomas, 1992), and this proportion is expected to expand further due to climate change (Huang et al., 2016). These regions are severely affected by harsh environmental conditions such as water shortages, high solar radiation, fluctuating temperatures, soil salinity and nutrient deficiencies, all of which significantly affecting plant growth and survival (Alsharif et al., 2020). To achieve the desired level of plant growth and crop production under these environmental conditions, using region-specific microbial strains as inoculum is a promising alternative to chemical fertilizer. For example, *Bacillus cereus* and *Bacillus albus*, two PGPBs from arid regions, possess distinct characteristics that enable them to thrive in extreme environments, including low nutrient availability, high salinity, intense solar radiation, and drought (Ashry et al., 2022; Patani et al., 2023). Our recent study futher demonstrates this potential: genome characterization and greenhouse trials with *Bacillus* sp. SW7 isolated from arid mangroves, showed multiple PGP related genes and enhanced tomato plant growth (Afridi et al., 2024).

Worldwide, PGPBs reported to enhance the growth and production of many crops such as maize, wheat and tomatoes (Ricci et al., 2019; Prudêncio de Araújo et al., 2020; Stella et al., 2025; Xu et al., 2022). Tomatoes are among the most widely used vegetables globally (Toni et al., 2021). Increased production, protection, and quality of tomatoes require non-chemical alternatives. Studies with PGP *Bacillus*-based biofertilizers have shown that they enhance tomato production and fruit quality. Several *Bacillus* species, including *Bacillus licheniformis, Bacillus subtilis, Bacillus cereus, Bacillus amyloliquefaciens, Bacillus megaterium, Bacillus* sp. SW7, *and Bacillus pumilus* successfully colonize tomato rhizospheres and enhance growth and production (Afridi et al., 2024; Akram et al., 2019; AlHosani et al., 2025; Du et al., 2022; Luo et al., 2022; Samaras et al., 2021; Saxena et al., 2020; Yan et al., 2003).

In this study, we characterized the genome of *Bacillus* sp. SW14 isolated from mangrove sediments in Umm Al Quwain, United Arab Emirates (UAE) (25°32’06.0”N, 55°37’50.9”E) (Tounsi-Hammami et al., 2024). *Bacillus* sp. SW14 possesses multiple PGP and heat and saline stress-tolerating genes, among others. However, the strain’s genome characterization and field evaluation hadn’t been evaluated before. Therefore, we aimed to (i) characterize previously isolated *Bacillus* sp. SW14 using whole genome sequencing to identify gene clusters responsible for PGP traits and (ii) perform pangenome and phylogenomic analysis to understand the taxonomic properties of *Bacillus* sp. SW14; and (iii) evaluate the effect of *Bacillus* sp. SW14 on the seed germinations and growth of tomato (*Solanum lycopersicum*) in the greenhouse experiment.

## Materials and methods

### Seed preparation, surface coating, and germination experiment

Tomato seed viability was estimated using the float test (Degwale et al., 2023). Tomato seeds (*Solanum lycopersicum*) were collected and coated as described earlier (Mahdi et al., 2020). Briefly, seeds were surface sterilized for 1 min using 5% sodium hypochlorite solution, followed by rinsing with 70% ethanol for 1 min. The seeds were then rinsed using sterilized distilled water 5 times and air-dried in a laminar hood. *Bacillus* sp. SW14 was inoculated in Lura Bertani (LB) broth and incubated at 30°C for 8 h. The bacterial pellets were then separated using centrifugation at 10,000 rpm for 5 min (when OD_600_ nm = 0.8), and the bacterial cells were coated onto the seeds following the method described earlier (Afridi et al., 2024; Mahdi et al., 2020). After coating, the seeds were then air-dried and placed on sterile filter paper in petri plates with 30 seeds per plate, this method was used to test germination and greenhouse trial. All the treatments were maintained in triplicates, and the seeds treated with distilled water served as a control. Germination rates in petri plates were measured after 14 days of incubation at 25°C in the dark. The total seedling length, as well as fresh and dry weights, were measured.

According to the following equations, the germination rate was calculated (Islam et al., 2016) (Equation 1):


(1) germination rate (%) = (Number of germinated seeds/total seeds) × 100%)

### Greenhouse trial

Sowing preparations began as soon as the seeds were coated. We prepared 50 pits in each potting tray for seedlings. Before starting the sowing process, the trays were labelled according to treatment and placed in the greenhouse at the International Centre of Biosaline Agriculture (ICBA), Dubai, UAE. In the greenhouse, conditions were similar to those described by (Rath and Ghosal, 2020). Using sterilized forceps, each seed was picked up and planted in each pit using small dents in the soil. We then irrigated the trays and left them to germinate. We watered the trays 3 times daily to keep the soil moist, which is optimal for seed germination. Irrigation was slightly increased as seedlings began to grow.

In the greenhouse, after monitoring seedling growth for 4 weeks and collecting data, the seedlings that were in good shape were transplanted directly from the potting trays into the greenhouse, where they received almost constant irrigation for another 4 weeks until they reached a mature size. Greenhouse conditions were kept similar as described by (Quamruzzaman et al., 2021). Greenhouse conditions were not strictly controlled; instead, they were determined by the weather, temperature, and humidity of the day. We used saganizer garden hand to create minor perforations in the soil, subsequently supplemented with a blend of NPK fertilizer and organic humic substance at a ratio of 1:3 added. This practice was undertaken to enhance the germination and subsequent growth of seedlings, as well as to facilitate the optimal development of transplanted seedlings within the confines of a greenhouse field trial environment. The seedlings were watered twice a day, once in the morning and once in the evening, for the weeks as described earlier (Ashry et al., 2022; Patani et al., 2023).

### Assesment of *Bacillus* sp. SW14 on plant physiological parameters

SPAD analyses were performed to evaluate the nutritional status of fully matured leaves, with three replicates per plant and five plants per group, utilizing a 502P chlorophyllometer (Konica Minolta, Inc., Tokyo, Japan). Leaf area index (LAI) was determined by measuring leaf length (L) and width (W) and applying the formula (L × W × 0.75), where 0.75 denotes the leaf compensating factor. Three leaves per plant were collected, and water content was assessed by determining fresh (Fw) and dry (Dw) weights. Dry weight (Dw) was measured after incubation at 104°C for two h and at 80°C for 72 h. Water content (%) was calculated using the formula: (Fw−Dw)/Fw∗100. Leaf density was determined using the gravimetric method, which involves dividing leaf mass by leaf area, typically denoted in grams per square meter (g/m²). For chlorophyll analysis, three leaves per plant were collected and frozen at -20°C. 0.2 g of frozen leaf samples were mixed with 4 ml of dimethyl sulphoxide for 24 h, followed by absorbance measurements at wavelengths of 663 nm, 645 nm, and 470 nm. Chlorophyll a, chlorophyll b, total chlorophyll (chlorophyll a + b), and carotenoid contents were calculated using the equations reported by (Batool et al., 2020). Statistical comparisons between control and inoculated samples were conducted using the Student’s t-test, with significance determined at P < 0.05.

### DNA extraction, and whole genome characterization

We used a Qiagen power soil DNA kit for genomic DNA isolation by following the manufacture protocol. The whole genome sequencing was performed using Oxford Nanopore technology (ONT) and short read (MGI). For the ONT, the library was constructed using Ligation sequencing kit V14 (SQK-LSK114) with the PCR-96 expansion kit, and the sequence was done in MinION MKC140 using an R10.4.1 flow cell (in-house). The resulting pod5_pass files were subjected to base-calling using High accuracy base-calling (DAN-400bps -5kHz), which removes barcodes and ONT adapter from the sequence, resulting in a trimmed sequence. For MGI sequencing, the library was constructed following our previous study (Afridi et al., 2024) and sequenced using an MGI sequencer (150 bp x 2 paired-end) in BGI-China. The MGI raw reads quality was assessed using the FastQC V 0.12.1 tool (Brown et al., 2017) and trimmed using Trimmomatic (V 0.39) to remove low-quality reads (with a Phred score below 20) with the following parameters of ILLUMINACLIP: TruSeq3-PE. fa: 2:30:10:2: True LEADING:3 TRAILING:3 SLIDINGWINDOW: 4:20 MINLEN:36 (Bolger et al., 2014). We adapted hybrid genome assembly using Unicycler (V 0.5.1) using the trimmed ONT long reads and MGI short reads using standard parameters (Wick et al., 2017). The quality of assembly was assessed using Quast 5.2.0 (Gurevich et al., 2013) and CheckM (Parks et al., 2015). and Busco (version is 4.1.4) using bacteria_odb10 (Simão et al., 2015). Followed by annotation of assembly using Prokka V 1.14.6 (Seemann, 2014) and prodigal (Hyatt et al., 2010). Further, we also used the open web-based platform PLaBase-PGPT-Pred (https://plabase.cs.uni-tuebingen.de/pb/form.php?var=PGPT-Pred) to identify PGP genes (Patz et al., 2021). In addition, we identified plant-bacteria interaction genes and their mechanisms using PLaBAse-PIFAR (https://plabase.cs.uni-tuebingen.de/pb/form.php?var=PIFAR-Pred) (Patz et al., 2021).

The Prokka format protein (amino) sequences of the assembled genome were used for functional annotation using the COG classifier (https://pypi.org/project/cogclassifier), and we used the Kyoto Encyclopaedia of Genes and Genomes database and blastkoala to identify the pathways. The assembled genome, along with the predicted genes non-coding tRNA sequences, were used to create a circle genome map using the Circos version 0.69-6 (Krzywinski et al., 2009). Both the ONT and MGI reads, along with the assembly, were deposited in the National Centre of Biotechnology Information (NCBI) database under the accession number PRJNA1068844 under Biosample SAMN46135429 (SRR31926110 and SRR31926111) and assembled genome under the accession JBLKRW000000000.

### Pangenome and phylogenomic analysis

We conducted pangenome and phylogenomic analysis using anvio (V8) (Eren et al., 2020), for these phylogenetically closest genomes were downloaded (based on the Typer Strain server), along with *Bacillus* sp. that reported as PGPB in semi and hyper-arid regions. Overall, 15 closely related *Bacillus* genome were used, i.e. *Bacillus altitudinis* strain 19RS3 (JACAAH010000008.1), *Bacillus altitudinis* strain GLB197 (CP018574.1), *Bacillus altitudinis* strain GQYP101 (NZ CP040514.1), *Bacillus altitudinis* strain T5S-T4 (JACAAI010000042.1), *Bacillus altitudinis* strain W3 (NZ CP011150.1), *Bacillus amyloliquefaciens* subsp (CP000560.2), *Bacillus anthracis* str Ames (AE016879.1), *Bacillus cereus* ATCC 14579 (CP034551.1), *Bacillus cereus* (CP017060.1), *Bacillus cereus* Rock4-18 (CM000735.1), *Bacillus cereus* strain T4S (NZ JAFNAY010000100.1), *Bacillus* sp. RZ2MS9 (CP049978.1), *Bacillus* sp. S1 R2T1-FB (NBNU01000100.1), *Bacillus subtilis* strain YB-15 (CP092631.1) and *Bacillus thuringiensis* YBT-1518 (CP005935.1). The pangenome was constructed following anvio (V8) bacterial pangenomics workflow – https://merenlab.org/tutorials/vibrio-jasicida-pangenome/ (Delmont and Eren, 2018). In detail, the genomes were processed through the following scripts: anvi-script-reformat-fasta (to convert the fasta to anvio readable format), then anvi-gen-contigs-database used to create a database, which was then combined, future used for identifying open reading frames using Prodigal v2.6.33 (Hyatt et al., 2010). Then, we calculate the similarity between the genomes using pyANI (anvi-compute-genome-similarity). We compute the pangenome using anvi-pan-genome and visualized using anvi-display-pan. The circular *Bacillus* sp. SW14 genome with prokka annotations were created using Anvi’o, which utilizes MUSCLE (Edgar, 2004) for sequence alignment. The annotation were imported with anvi-import-functions, followed by profiling with anvi-profile, and visualized using anvi-interactive. We performed a comparative analysis to identify shared and unique genes between *Bacillus* sp. SW14 and its closest relative, *Bacillus vallismortis* DV1 F-3. Orthologous gene clusters were identified using OrthoVenn3 (Sun et al. 2023) with the amino acid sequences from their Prokka-annotated genomes as input. This analysis enabled us to determine the unique genomic content of strain SW14.

## Results

### Seed germination and greenhouse experiment

We observed a slight improvement in the germination rate of tomato seeds treated with the strain SW14 compared to the control group, though the difference was not statistically significant. Furthermore, greenhouse trials revealed improvements across various parameters, indicating the positive impact of the SW14 strain on tomato plant growth and development. Notably, a significant difference was observed in leaf dry weight (P = 0.047), leaf surface area (P = 0.0132), carotenoids (P = 0.0029) and total chlorophyll and carotenoid ratio (P = 0.0101) (**Table 1**). Other parameters, such as SPAD value, also increased from the control group to the strain SW14 inoculated group (**Table 1**). The number of leaves per plant and shoot height were also increased in the SW14 strain inoculated group compared to the control group, indicating enhanced leaf development. However, no significant difference was observed in chlorophyll a and b levels between SW14 inoculated and control plants. Overall, the results suggest that the SW14 strain significantly improved plant growth traits and chlorophyll contents compared to the untreated control plants.

**Table 1:** Effect of Bacillus sp. SW14 inoculation on various plant growth and physiological parameters.

Variable	Treatment	Mean ± SD	t-value	p-value
Germination rate	control	10.67 ± 0.94	1.3416	0.2508
	Inoculated	11.67 ± 0.27		
SPAD	control	31.01 ± 1.46	2.1272	0.1005
	Inoculated	33.79 ± 1.68		
Leaf number	control	6.82 ± 2.31	1.6193	0.1807
	Inoculated	20.51 ± 11.55		
Leaf fresh weight	control	0.011 ± 0.044	1.2319	0.2855
	Inoculated	0.015 ± 0.0035		
Leaf dry weight	control	0.0024 ± 0.00066	2.8284	0.0474*
	Inoculated	0.0045 ± 0.0013		
Leaf water content	control	0.00011 ± 0.000034	0.5682	0.6003
	Inoculated	0.00010 ± 0.000017		
Leaf surface area	control	1.8233 ± 0.144	4.2457	0.0132*
	Inoculated	15.15 ± 4.13		
Shoot height	control	4.56 ± 0.94	0.0851	0.085
	Inoculated	10.54 ± 3.44		
Chlorophyll a	Control	2.21 ± 0.84	1.8372	0.1401
	Inoculated	3.39 ± 0.51		
Chlorophyll b	Control	0.79 ± 0.39	1.6097	0.1827
	Inoculated	1.18 ± 0.09		
Total chlorophyll	Control	3.33 ± 1.26	1.8155	0.1436
	Inoculated	4.91 ± 0.58		
Carotenoids	Control	0.81 ± 0.29	8.996	0.0029*
	Inoculated	4.70 ± 0.55		
Total Chlorophyll and carotenoids	Control	3.81 ± 1.38	4.5891	0.0101*
	Inoculated	9.27 ± 1.28		

The table presents the mean and standard deviation for both control (un-inoculated) and inoculated plants. The statistical comparison between the two groups was performed using a t-test, with the corresponding p-values also represented. An asterisk (*) indicates a statistically significant difference between the control and inoculated groups (p < 0.05).

### Genome characteristics

**Figure 1:**
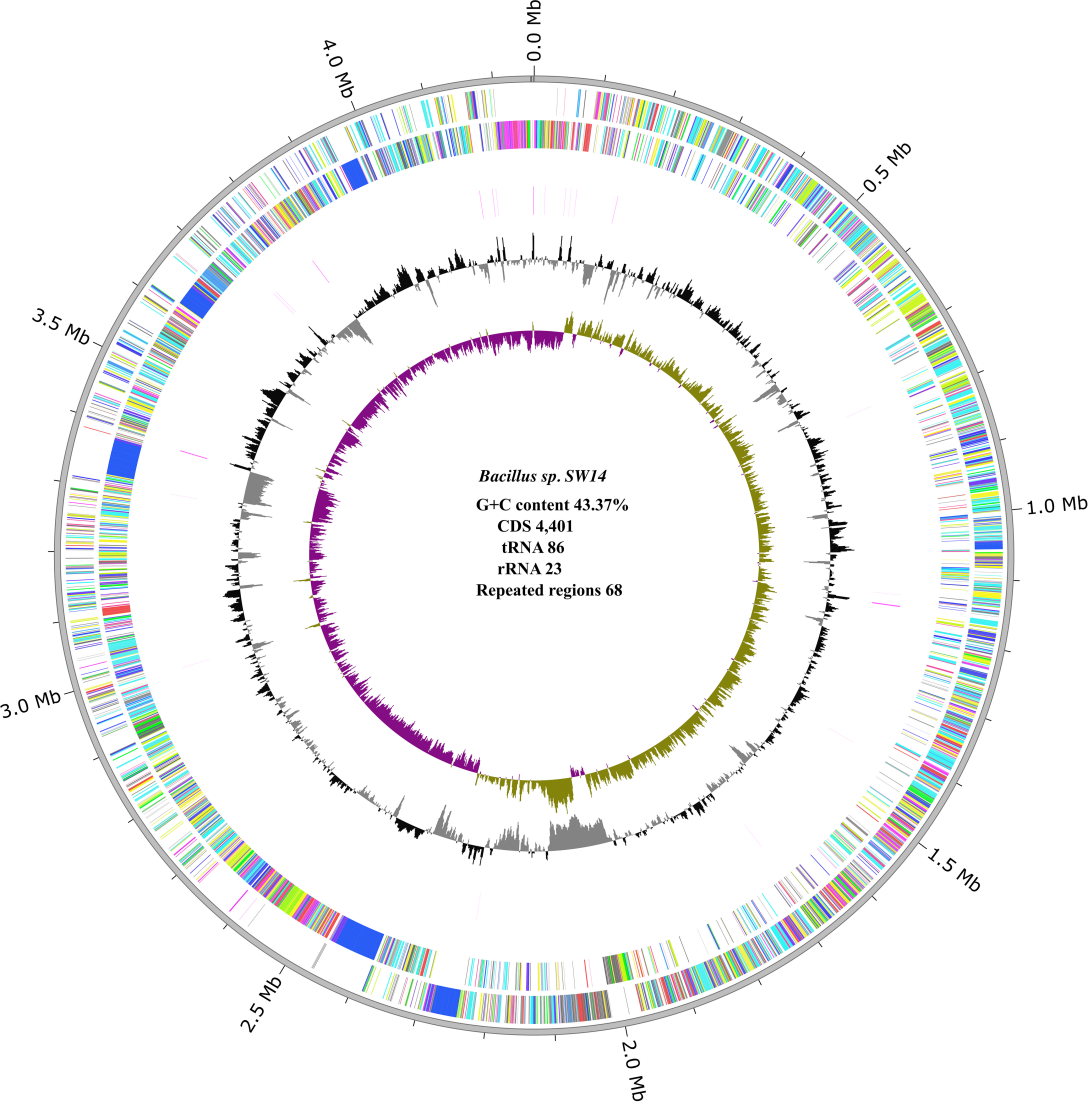
Circular genome representation of *Bacillus* sp. SW14, the innermost violet and green circle represents the GC Skew (+ and -), followed by black (+) and grey (-) circles representing GC content. The outermost circle represent the forward CDS, followed by the reverse CDS.

The assembled genome of strain SW14 was 4.25 Mb in size, with 100% completeness and 0.02% contamination, and a G+C content of 43.72% (**Figure 1**). Busco analysis also confirmed the genome’s completeness, identifying 124 single-copy genes without duplication. The strain SW14 genome encoded 4,401 CDS, 86 tRNA, 23 rRNA, and 68 repeat regions. Of the predicted proteins, 3,618 were assigned to proteins with function assignments, while 783 were classified as hypothetical proteins. Additionally, 1,058 proteins were assigned enzyme commission (EC) numbers, 880 proteins were annotated with GO terms, and 773 proteins were linked to pathway functions. A total of 80.25% of genes were assigned to COG function categories. Among these, 294 genes were associated with carbohydrate transport and metabolism, 289 with amino acid transport and metabolism, 277 with transcription, and 227 with translation, ribosomal structure and biogenesis. Detailed information on the protein assigned to COG categories is in a **Supplementary File** (**Supplementary Figure S1**).

### House-keeping genes-based phylogenetic analysis

**Figure 2:**
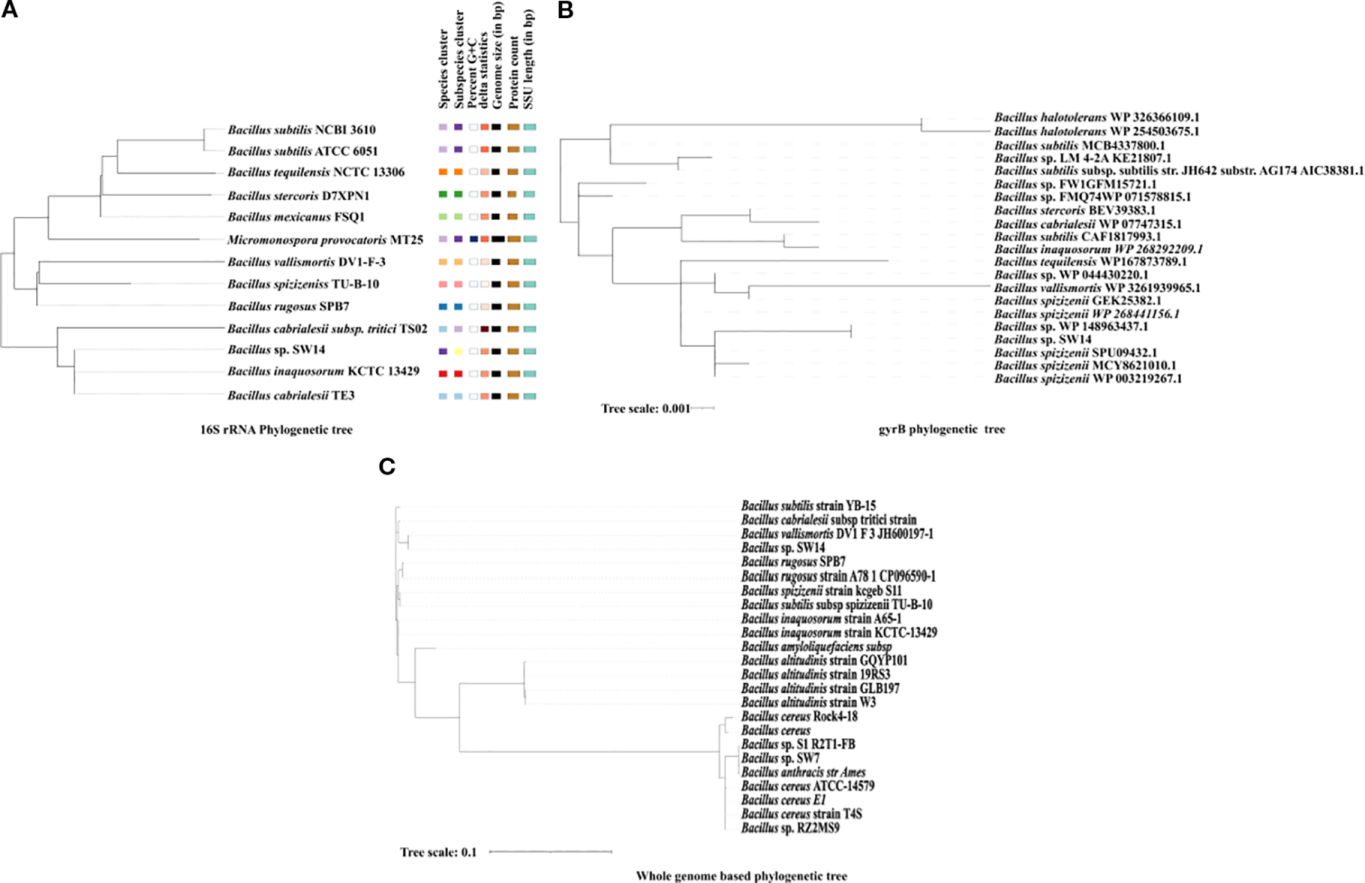
**(A)** 16S rRNA-based phylogenetic tree generated using the Type strain server, showing the phylogenetic relationship of *Bacillus* sp. SW14 with its closely related species. The tree includes genomic features such as G+C content, genome size, protein count and SSU length. **(B)** gyrB-based phylogenetic tree, constructed using the housekeeping gene gyrB, demonstrates *Bacillus* sp’s evolutionary placement. The strain shows a close phylogenetic relationship with *Bacillus* species based on gyrB gene similarity. **(C)** A whole genome-based phylogenetic tree was created using a Anvi'o comparing *Bacillus* sp. SW14, with closely related *Bacillus* genomes and other previously reported PGP *Bacillus* species, showed that *Bacillus* sp. SW14 is closely related to *Bacillus Vallismortis*.

The phylogenetic test based on the 16S rRNA showed that the strain SW14 was more closely related to *Bacillus inaquosorum* KCTC 13429 and *Bacillus cabrialesii* (**Figure 2A**). However, based on the housekeeping gene gyrB, the strain SW14 was closely associated with multiple unclassified *Bacillus* species, which suggests that SW14 represents a novel strain (**Figure 2B**).

### Whole genome-based phylogenomics average nucleotide identity and pangenomics

**Figure 3:**
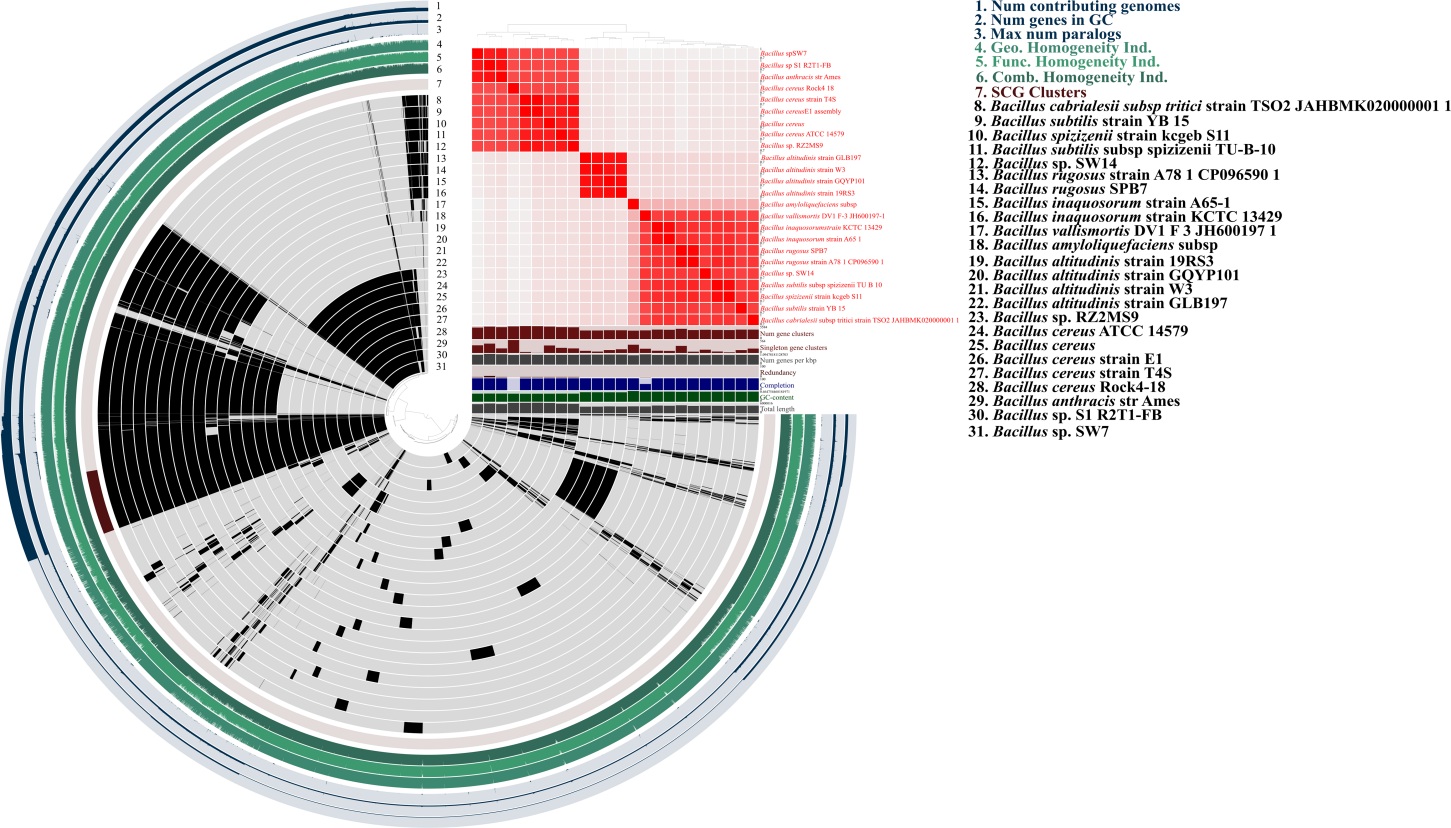
Comparative pangenome and ANI analysis insides into novel *Bacillus* sp. SW14 and its closely related *Bacillus* species genomes. Pangenome analysis elucidate shared (black) and unique (gray) proportion of gene clusters with *Bacillus* sp. SW14. The pangenome also represents gene clusters, singleton genes, completeness, GC content *homogeneity, single-copy gene (SCG) cluster, Kofam and KEGGs*. Also, ANI shows that SW14 shares only ~ 93% similarity to its closest available genome in the NCBI and the Type strain server database.

The type strain server identified the strain SW14 as a potentially novel species, as no closely related genome was available in the database. In the phylogenomic tree, the strain SW14 formed a distinct branch, separate from other *Bacillus* species, though it was most closely related to the *Bacillus vallismortis* DV1 F-3 strain (**Figure 2C**). Based on pyANI analysis, the genome of strain SW14 exhibits only 93.58% similarity to *Bacillus vallismortis* DV1 F-3, followed by 93.3% similarity to *Bacillus* species, including *Bacillus inaquosorum* strain A65-1, *Bacillus inaquosorum* strain KCTC 13429, *Bacillus rugosus* SPB7, *Bacillus rugosus* strain A78–1 and *Bacillus subtilis subsp* sp*izizenii* TU-B-10. Furthermore, the genome of strain SW14 shared only 71-73% homology to other *Bacillus* species (**Figure 3**). A comparison of *Bacillus* sp. SW14 and its closest relative, *Bacillus vallismortis* DV1 F-3, revealed a distinct genomic content for our strain. *Bacillus* sp. SW14 harbored 36 unique clusters containing 791 singleton genes, while *Bacillus vallismortis* DV1 F-3 had 27 unique clusters with 703 singletons, both the strains shared 3,250 gene clusters. These unique genes likely contribute to the unique PGP traits and environmental adaptation observed in *Bacillus* sp. SW14.

### Plant growth-promoting traits related gene

**Figure 4:**
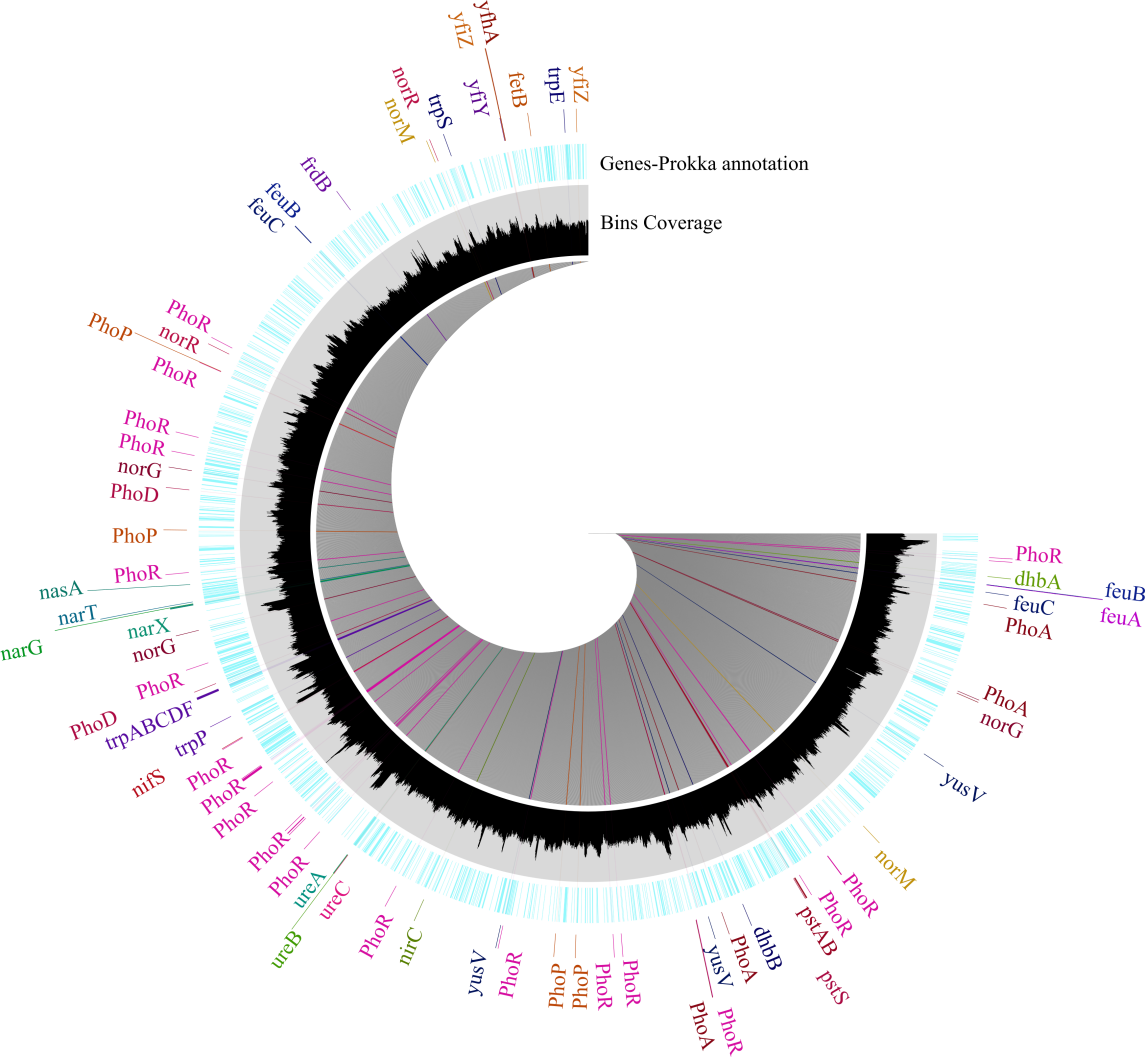
Circular representation of Prokka-annotated functional gene of *Bacillus* sp. SW14. The outer (blue) ring display gene annotation using Prokka, with each gene represented in a distinct color to indicate different functional categories, including PGP genes. The inner (black) ring shows the distribution of genes organized into bin using Anvi’o.

Based on PGPT_pred analysis, we identified several PGP genes associated with nitrogen fixation, like *nifSUM*, whereas only *nifS* was detected using Prokka annotation (**Figure 4**). Prokka annotation identified that the genome of strain SW14 consists of genes involved in denitrification and nitrate reduction (*narGTHX*, *nasA, nfrA, nirC*, and *norQRM*), whereas PGPT_pred identified more number of genes (**Supplementary File 2**) as well as nitrate and nitrite transport (*nirC*), urea metabolism and transport (*ureABC*) were also identified in both annotation, in addition to this genes such as *urtABCDE* involving in urea transport were identified using PGPT_pred. Phosphate solubilization metabolism (including transport) genes, such as *pqq*, *PhoADEHPRU*, and *pstABCS*, were identified based on PGPT_pred (**Supplementary File 2**). However, only *phoABDR* and *pstABS* were identified via Prokka analysis (**Figure 4**). Likewise, potassium solubilizing genes, including *kch/mthK, trkAGH* and *ktrAB*, were identified using PGPT_pred. Siderophore-related genes (*dhbABCEF*) and their supporting transport mechanisms genes like *yusV, yfhAZ, yflTY*, and *yfiZ* were also present in the SW14 genome, also identified genes associated with iron transport genes (*fetBD* and *feuABC*). Both annotation identified tryptophan precursor genes responsible for IAA synthesis (*trpABCDEFS*). However, the direct IAA-producing genes, such as *IpdC* and *dhaS*, were absent. Interestingly, based on PGPT_pred, the IAA pathway genes like iaaT/yedL/ysnE and yhcx were identified (**Supplementary File 2**).

### Environmental stress and bioprospecting related genes

Apart from PGP genes, major heat shock proteins (*GrpE, dnaJ, K and groL, S*), drought-resistant genes like *yciG* and *yxaL* (based on PGPT_pred), and salt tolerance gene (*RelA*), were identified. Gene traits for secret bactericidal compounds, e.g. aurachin, bacillaene, rifamycin, bacteracin, cephalosporin, cycloserine, nocardicin, prodigiosin, spermidine, surfactin, toxoflavin, (*acpP, acpK, baeDHLSEGMRCN, cvpA, rseP, ybhFS, cah, dcsA, mbtH, fabDI, paiA*, sp*eBEG, srfAA, srfab, srfAC, ribD*, and *toxF*) (**Supplementary File 2**) and fungicidal compounds biosynthesis genes like *bpsAB*, asm14,17, *tktAB, ituB*, *cbpS*, *nagZ*, *ppsABCD, ntdABC*, and *toxF* were identified in the SW14 genome (**Supplementary File 2**).

## Discussion

*Bacillus* sp. are widely recognized for their ability to promote plant growth and suppress the development of phytopathogens, making them a promising alternative to biopesticides and biofertilizers. Studies indicate that nearly half of the commercially available biocontrol bacterial products are based on *Bacillus* strains (Zhang et al., 2023). To assess the plant growth-promoting potential of *Bacillus* sp. SW14, we examined its effects on tomato phenotypic and physiological traits, alongwith genomic determinants underlysing these functions. Our findings show that *Bacillus* sp. SW14 enhance plant growth traits such as seed germination, plant leaf number, leaf water content, leaf area index, shoot height, SPAD, and chlorophyll contents. Further genome analysis reveals multiple PGP and stress-related genes in this strain, rendering it a potentially useful PGP bacterium for arid environments. Phylogeny and pangenome analysis show the *Bacillus* sp. SW14 is a novel strain closely related to *Bacillus vallismortis*.

### *Bacillus* sp. SW14 improved tomato seed germination and plant growth traits

We found that the strain SW14 significantly enhanced tomato seed germination and growth. This enhancement was reflected in various physiological traits, including SPAD, leaf number, leaf surface area, leaf fresh weight, leaf dry weight, leaf water content, shoot height, and chlorophyll content. Our findings are consistent with previous studies showing that the mangrove sediments associated bacterium *Bacillus* sp. SW7 strains significantly enhanced plant growth in tomato plants (Afridi et al., 2024). Several other *Bacillus* species like *Bacillus licheniformis, Bacillus cereus, Bacillus safensis*, and other *Bacillus* sp. have been reported to improve seed germination in arid and saline environments (Mahmoud et al., 2024; Mukhtar et al., 2023; Patani et al., 2023). In our result, increased seed germination was observed in the SW14 strain inoculated group compared to the control. Due to the ability of PGPBs to colonize seeds and produce lytic enzymes that break down seed coats, bacterial entry into the seeds is facilitated. Additionally, PGPBs synthesis phytohormones that regulate seed germination (Pérez-García et al., 2023).

Furthermore, PGPBs produce biomolecules such as antibiotics, siderophore, and hydrogen cyanide, which align with our early study, showing that the SW14 strain produces IAA (8.55 ppm), siderophore (7.67 ± 2.31), and hydrogen cyanide (Tounsi-Hammami et al., 2024). Additionally, the genome analysis shows the presence of tryptophan precursor genes responsible for IAA synthesis and siderophore genes. Our results were consistent with other PGPB strains, such as *Acinetobacter radioresistens* KBENdo3P1 (Pérez-García et al., 2023) and *Bacillus* sp. SW7 (Afridi et al., 2024) shown to have multiple PGP traits.

Under the greenhouse conditions, the *Bacillus* sp. SW14 strain inoculated tomato plants showed enhanced plant growth, especially on the leaf surface, leaf dry mass, and photosynthetic pigments, including carotenoids and total chlorophyll and carotenoid ratio. This is consistant with other studies where *Bacillus* sp. strains significantly improved plant growth traits in variuos crops under salt stress, respectively (AlHosani et al., 2025; Ali et al., 2022; Ayaz et al., 2021). This increase in photosynthetic pigments would be attributed to increased nutrient and water uptake, along with the production of extracellular polysaccharides and phosphate solubilization traits of PGPBs (Joshi et al., 2020; Kang et al., 2015). Our observations are in accordance with other studies that *Bacillus subtilis and B. amyloliquefaciensc*, *Enterobacter* sp. and *Pseudomonas fluorescens* increased chlorophyll content in tomato and wheat, respectively (Gashash et al., 2022; Singh and Jha, 2016). Moreover, some *Bacillus* sp. under drought conditions enhanced the photosynthetic activity by increasing magnesium and calcium, which regulates chloroplasts (Kamrul Huda et al., 2013). Furthermore, Katsenios et al. (2021) demonstrate that *Bacillus licheniformis* treatment increased the tomato dry weight, which is similar to our present study where leaf dry mass was higher in strain SW14 inoculated plants compared to control plants. This enhanced growth observed in strain SW14 inoculated tomato plants is attributed to the presence of plant growth-promoting genes, present in the SW14 strain. Together, these findings suggest that inoculation with specific *Bacillus* sp. can be an effective approach to improve tomato seed germination and overall plant growth traits, particularly in challenging arid environments.

### Plant growth promoting genes in SW14

The strain SW14 includes nitrogen fixing ability, which is essential for the plants to grow, and the genome confirmed the presence of nitrogen-fixing genes like *nifSUM*, which are involved in Fe-S cluster assembly, required for the activation of nitrogenase. Several PGP *Bacillus* species, such as *Bacillus halotolerant* and *Bacillus amyloliquefaciens* known to have only *nifS* and *nifU* genes (Pinto et al., 2018; Wang et al., 2023). Even though key nitrogen genes were not identified in the assembled genome, genes involved in denitrification, nitrate/nitrite transport, nitrogen regulation, ammonia production and urea metabolism were present, reflecting the strain role on nitrogen metabolism (Sprent and de Faria, 1988). Similar genome structures except key nitrogen fixing genes have been observed in *Bacillus altitudinis* FD48 (Narayanasamy et al., 2023) and *Bacillus amyloliquefaciens* subsp. plantarum strain Fito_F321 (Pinto et al., 2018). We also identified the gene Pyrroloquinoline quinine (*pqq*) in the genome, which involved P solubilization along with a complete set of assimilation and transport genes were identified. Most reported PGP *Bacillus* species have either P transport or assimilation (Cortese et al., 2021; Xu et al., 2022; Juby et al., 2024). To overcome osmatic stress, plants need potassium, and PGP bacteria aid by solubilizing and transporting K, which helps plants mitigate osmatic stress. We identified a gene responsible for K solubilization, uptake and transport in the SW14 genome, consistent with other reported PGP *Bacillus* species (Chen et al., 2022; Xu et al., 2022; Narayanasamy et al., 2023). Additionally, indirect support mechanisms, like siderophore production, were evident in the SW14 genome. Gene clusters responsible for iron uptake and bacillibactin biosynthesis were identified which are similar with other PGP *Bacillus* species, *Bacillus altitudinis* T5S-T4 (Cortese et al., 2021), *B. subtilis* MBB3B9 (Hazarika et al., 2023), *B. altitudinis* FD48 (Narayanasamy et al., 2023), where these genes play key role in iron acquisition and promotes plant growth.

Plant hormones produced by the PGP bacteria, such as IAA, gibberellic acids and cytokinin, have a significant role in plant growth promotion. Bacteria isolated from the rhizosphere are mostly able to synthesize IAA from tryptophan through five different pathways. Even though the strain SW14 genome has no direct genes such as *ipdC* and *iaaH* involved in IAA production, it consists of a complete set of genes such as *iaaT* and *yhcX* as well as tryptophan pathway genes (*trpABCDEFS*) which conform the *in-vitro* produce of IAA the strain SW14 (Tounsi-Hammami et al., 2024). Similarly, the presence of only tryptophan genes, a precursor for IAA, was noted in other *Bacillus* spp., such as *Bacillus* sp. IHBT-705 (Ali et al., 2023), and *B*. *altitudinis* 19RS3 and *B*. *altitudinis* T5S-T4 (Cortese et al., 2021).

## Conclusion

This study presents the complete genome sequence and greenhouse assessment of the multifunctional *Bacillus* sp. strain SW14 as a PGPB. The *in-vitro* screening findings were validated through genome analysis, supporting the strain SW14 as a PGPB. The strain harboured numerous signature genes associated with PGP traits in tomatoes, including indole-3-acetic acid (IAA) production, siderophore production, ammonia (NH_3_) production, as well as phosphate and potassium solubilization. These genetic attributes highlight the capacity of *Bacillus* sp. SW14 to enhance tomato plant growth. Additionally, the genome contains heat and salinity-tolerant genes, suggesting that the strain is well-suited for arid environments. Furthermore, the greenhouse experiment demonstrated that *Bacillus* sp. SW14 enhances plant growth parameters such as SPAD, leaf dry mass, shoot height, Chlorophyll a and b, carotenoids and total chlorophyll and carotenoid ratio. A more comprehensive comparative analysis might provide more insights into the mode of action of PGPs on their eukaryotic hosts under greenhouse conditions. However, future research is needed to assess the molecular mechanisms of *Bacillus* sp. SW14 under less controlled field-based environments to validate its efficacy in arid agriculture settings.

## Data Availability

The datasets presented in this study can be found in online repositories. The names of the repository/repositories and accession numbers can be found in the article/**Supplementary Material**.
