# Supplementary material for: Bacillus sp. SW14 isolated from arid mangroves sediments enhances tomato plant growth: insights from genome analysis and greenhouse evaluation
Source: Front Plant Sci. 2025 Oct 8;16:1673790. doi: 10.3389/fpls.2025.1673790 (PMC12542870; doi:10.3389/fpls.2025.1673790)
Supplement: Supplementary file 2 [file DataSheet1.docx]

**Supplementary file for**

***Bacillus* sp. SW14 Isolated from Arid Mangroves sediments Enhances Tomato Plant Growth: Insights from Genome Analysis and Greenhouse Evaluation**

Balamurugan Sadaiappan^1^, Qurban Ali^2^ , Mahideen Afridi^2,3^, Munawwar Ali Khan^4^, Sunil Mundra^1,2,5*^

^1^ Khalifa Center for Genetic Engineering and Biotechnology (KCGEB), United Arab Emirates University, Al Ain, United Arab Emirates

^2^ Department of Biology, College of Science, United Arab Emirates University, Al-Ain, Abu-Dhabi, UAE

^3^ National Nanfan Research Institute, Chinese Academy of Agriculture Sciences, Sanya 572024, China

^4^Department of Life and Environmental Sciences, College of Natural and Health Sciences, Zayed University, Dubai, UAE

^5^National Water and Energy Center, United Arab Emirates University, Al Ain, United Arab Emirates

**^*^**Corresponding author: [sunilmundra@uaeu.ac.ae](mailto:sunilmundra@uaeu.ac.ae)

+971-03-7136341


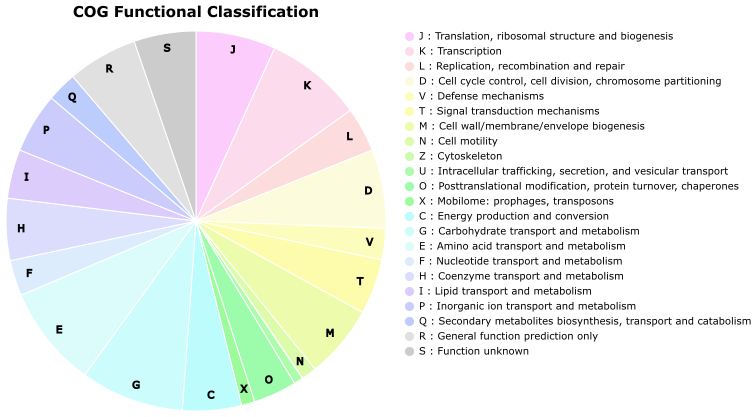


Figure S1. The pie-chart represents the **Clusters of Orthologous Groups (COG) classification** predicted for *Bacillus* sp. SW14 genome. Each segment in the pie chart represents a functional category, labeled by a **COG letter** and its description.

| **LETTER** | **COUNT** | **DESCRIPTION** |
| --- | --- | --- |
| **J** | 227 | Translation, ribosomal structure and biogenesis |
| **A** | 0 | RNA processing and modification |
| **K** | 277 | Transcription |
| **L** | 127 | Replication, recombination and repair |
| **B** | 0 | Chromatin structure and dynamics |
| **D** | 224 | Cell cycle control, cell division, chromosome partitioning |
| **Y** | 0 | Nuclear structure |
| **V** | 89 | Defense mechanisms |
| **T** | 157 | Signal transduction mechanisms |
| **M** | 208 | Cell wall/membrane/envelope biogenesis |
| **N** | 43 | Cell motility |
| **Z** | 1 | Cytoskeleton |
| **W** | 0 | Extracellular structures |
| **U** | 26 | Intracellular trafficking, secretion, and vesicular transport |
| **O** | 123 | Posttranslational modification, protein turnover, chaperones |
| **X** | 38 | Mobilome: prophages, transposons |
| **C** | 167 | Energy production and conversion |
| **G** | 294 | Carbohydrate transport and metabolism |
| **E** | 289 | Amino acid transport and metabolism |
| **F** | 101 | Nucleotide transport and metabolism |
| **H** | 176 | Coenzyme transport and metabolism |
| **I** | 139 | Lipid transport and metabolism |
| **P** | 171 | Inorganic ion transport and metabolism |
| **Q** | 85 | Secondary metabolites biosynthesis, transport and catabolism |
| **R** | 199 | General function prediction only |
| **S** | 176 | Function unknown |
